# Circulating angiopoietin-like proteins in metabolic-associated fatty liver disease: a systematic review and meta-analysis

**DOI:** 10.1186/s12944-021-01481-1

**Published:** 2021-05-25

**Authors:** Yani Ke, Shan Liu, Zheyuan Zhang, Jie Hu

**Affiliations:** 1 The Second Clinical Medical College of Zhejiang Chinese Medical University, No 548, Binwen Road, Hangzhou, 310051 Zhejiang Province China; 2 Department of Clinical Evaluation Center, The First Affiliated Hospital of Zhejiang Chinese Medical University, No. 54, Youdian Road, Hangzhou, 310006 Zhejiang Province China; 3 The First Clinical Medical College of Zhejiang Chinese Medical University, No 548, Binwen Road, Hangzhou, 310051 Zhejiang Province China; 4 Department of Infectious Diseases, The First Affiliated Hospital of Zhejiang Chinese Medical University, No. 54, Youdian Road, Hangzhou, 310006 Zhejiang Province China

**Keywords:** Metabolic-associated fatty liver disease, Non-alcoholic fatty liver disease, Non-alcoholic steatohepatitis, Angiopoietin-like proteins, Angiopoietin-like protein 8, Meta-analysis

## Abstract

**Background:**

Angiopoietin-like proteins (ANGPTLs) are closely related to insulin resistance and lipid metabolism, and may be a key in metabolic syndrome. Non-alcoholic fatty liver disease (NAFLD) (newly named metabolic-associated fatty liver disease (MAFLD)) is based on metabolic dysfunction. There may be some correlation between ANGPTLs and MAFLD, but the specific correlation is unclear. This study aims to explore the predictive role of ANGPTLs in MAFLD and its progression.

**Methods:**

Seven databases (PubMed, EMBASE, Cochrane Library, CNKI, WANFANG, CBM and Clinicaltrials.gov) were searched with free terms and MeSH terms. The random-effects model was used to pool the data, and Standardized Mean Difference (SMD) and 95% confidence intervals (CI) were taken as the overall outcome. No language restrictions existed in the article selection. RevMan 5.3, Stata 16 and MetaXL software were applied to analyse the data and the GRADE system was utilized to assess the certainty of evidence.

**Results:**

After reviewing 823 related articles, 13 studies (854 cases and 610 controls) met the inclusion criteria, and contributed to this meta-analysis. The results showed that circulating ANGPTL8 level was significantly elevated in the MAFLD group than in the healthy control group (SMD = 0.97 pg/mL, 95%CI: 0.77, 1.18). Conversely, there was no significant difference in the ANGPTL4 (SMD = 0.11 ng/mL, 95%CI: − 0.32, 0.54) and ANGPTL3 (SMD = − 0.95 ng/mL, 95%CI: − 4.38, 2.48) between the two groups. Subgroup analysis showed that: 1) the MAFLD group had significantly higher ANGPTL8 levels than the healthy control group in Asian and other races; 2) the ANGPTL8 levels in Body Mass Index (BMI) > 25 kg/m^2^ patients with MAFLD were higher than those in the healthy control group; 3) the higher ANGPTL8 levels were observed in moderate to severe MAFLD group than the healthy control group. Meta-regression demonstrated that BMI might effectively explain the high heterogeneity. No significant publication bias existed (*P* > 0.05). The certainty of evidence was assessed as very low by the GRADE system.

**Conclusions:**

The ANGPTLs may be related to MAFLD. The increased ANGPTL8 level may be positively correlated with different situations of MAFLD, which may act as a potential indicator to monitor the development trends.

**Supplementary Information:**

The online version contains supplementary material available at 10.1186/s12944-021-01481-1.

## Background

Non-alcoholic fatty liver disease (NAFLD) has become a global concern as a consequence of aberrant obesity and unhealthy lifestyles [1]. It was re-named metabolic-associated fatty liver disease (MAFLD) in 2020 [2]. MAFLD is a sensitive and important indicator of metabolic dysfunction [3]. It is defined as a series of hepatic metabolic syndromes caused by hepatocyte steatosis [4] in more than 5% of the liver [5]. Owing to its high specificity and sensitivity, two-dimensional ultrasound has become the first choice for the clinical diagnosis of NAFLD. However, because of the insidious onset of NAFLD and the unawareness of its hazards, the rate of active consultation is low. Therefore, the diagnosis of NAFLD is rare in its early stages, and timely treatment is delayed. It is then necessary to find a convenient, efficient, and accurate laboratory diagnostic index for its detection.

Angiopoietin-like proteins (ANGPTLs) are a secreted glycoprotein family consisting of eight members, angiopoietin-like 1–8 (ANGPTL1–8) [6]. They share some common structures; however, there are also some specific structures that make them different in tissue expression and regulation [7–9]. Some studies have shown that ANGPTLs play an important role in insulin resistance, glucose metabolism, and hormone regulation [10–12]. Notably, ANGPTL8, also known as betatrophin, C19ORF80, or lipasin [13], regulates triglyceride metabolism by interacting with ANGPTL3 and ANGPTL4 to inhibit lipoprotein lipase (LPL) activity [14–16]. This further indicates that ANGPTLs are also closely related to lipid metabolism and may be an important link in metabolic syndrome [17–19].

An increasing number of studies have focused on the clinical relationship between ANGPTLs and NAFLD; however, their results are inconsistent. Therefore, the purpose of this study was to clarify whether there is a relationship between ANGPTLs and NAFLD and the mechanism underlying the relationship. Accordingly, the results may be helpful for the diagnosis of NAFLD and dynamic evaluation of disease progression. Therefore, a systematic review and meta-analysis was performed to obtain a more persuasive conclusion.

## Methods

### Search strategy

The entire process of this meta-analysis was performed in accordance with the PRISMA statement (see Supplementary Additional file 1). Furthermore, the protocol of this meta-analysis was registered in PROSPERO with number CRD42020159432 (see Supplementary Additional file 2).

Two researchers searched seven databases (PubMed, Cochrane Library, EMBASE, CNKI, WANFANG, CBM, and Clinicaltrials.gov) to find relevant articles published up to 2 April 2021. Free terms and MeSH terms, such as (‘MAFLD’ OR ‘metabolic-associated fatty liver disease’ OR ‘fatty liver’ OR ‘liver, non-alcoholic fatty’ OR ‘steatohepatitides, non-alcoholic’ OR ‘steatohepatitis, nonalcoholic’ OR ‘NASH’ OR ‘non-alcoholic fatty liver disease’ OR ‘NAFLD’ OR ‘nonalcoholic fatty liver disease’ OR ‘nonalcoholic fatty liver’ OR ‘nonalcoholic steatohepatitis’ OR ‘nonalcoholic steatohepatitides’) AND (‘angiopoietin-like protein’ OR ‘angiopoietin-like’ OR ‘ANGPTL’ OR ‘ANGPTLs’ OR ‘betatrophin’ OR ‘C19ORF80’ OR ‘lipasin’), were used to search for the relevant articles. In addition, the reference lists of articles with citations were also reviewed to identify any suitable papers. It was also necessary to contact authors by e-mail to clarify additional studies or ask for missing data. There were no language restrictions for the articles selected for the analysis.

### Study selection

Two researchers independently checked each study. If there was a dispute, a third researcher made reasonable judgments according to the protocol. Contacting authors by e-mail to obtain more detailed data was an important key to the study.

The inclusion criteria were as follows: 1) conducted in adults (aged ≥18 years); 2) the case group must be diagnosed with NAFLD (MAFLD) either by imaging or biopsy; 3) the control group must include healthy individuals without any metabolic diseases; 4) articles that focused on the circulating levels of ANGPTLs; and 5) case-control studies or cohort studies.

The exclusion criteria were as follows: 1) patients without NAFLD or NAFLD with other metabolic diseases; 2) factors related to secondary hepatic fat accumulation, such as alcohol consumption, use of liver injury medication, hereditary disorders, or other kinds of liver diseases; 3) other similar indices but not ANGPTLs; 4) ANGPTL levels in the liver, not in the plasma or serum; 5) non-comparison with healthy individuals; 6) case report, review literature, or animal experimental research; 7) repetitive articles; and 8) articles missing important data and no reply from the corresponding author.

### Data extraction and quality assessment

Two researchers independently extracted data, including the first author’s last name, publication date, country of origin, Newcastle–Ottawa Scale (NOS) score, ANGPTL level measuring method, numbers of cases and controls, basic information of cases and controls (such as age and sex), diagnostic methods, adjusted factors, levels of ANGPTLs in the NAFLD group/control group, and Body Mass Index (BMI) in the NAFLD group/control group. In addition, the grading of recommendation, assessment, development, and evaluation (GRADE) approach was used to evaluate the quality of our study from https://gdt.gradepro.org website.

### Statistical analysis

The Review Manager 5.3, Stata 16, and MetaXL software were used for the statistical analysis. The standardized mean difference (SMD) in the ANGPTL levels was used as the main outcome in this meta-analysis. Additionally, normally distributed data were presented as means ± standard deviations. To estimate the heterogeneities, the I^2^ test, Cochran’s Q-test, and Galbraith figure were used. The fixed-effects model was applied when there was unimportant heterogeneity (I^2^ < 40%) and the random-effects model when there was significant heterogeneity (I^2^ > 40%) [20–22]; a subgroup analysis together with a meta-regression analysis was conducted to explore the sources of high heterogeneity. If most of the included studies did not adjust basic information of the two groups, the robust error meta-regression would be chosen to reduce the influence of confounding factors [23], like BMI, severity and so on. Because of the non-negligible heterogeneity, the quality-effects model [24] was a good choice to address the impact of risk of bias on the effects.

The quality of the included articles was evaluated using the NOS score [25]. The overall quality score consists of three dimensions: assessment of selection, comparability, and exposure. A study can be awarded a maximum of one star for each numbered item within the selection and exposure categories. A maximum of two stars can be assigned for comparability. Egger’s test, funnel fig [26], Doi plot and the LFK index [27] were used to judge publication bias and investigate possible small study effects. Omission of each single study was applied for the purpose of sensitivity analysis, and Sensitivity to model selection was tested by using another model. The data form of mean difference (MD) was also utilised to measure sensitivity.

## Results

### Study selection

**Fig. 1:**
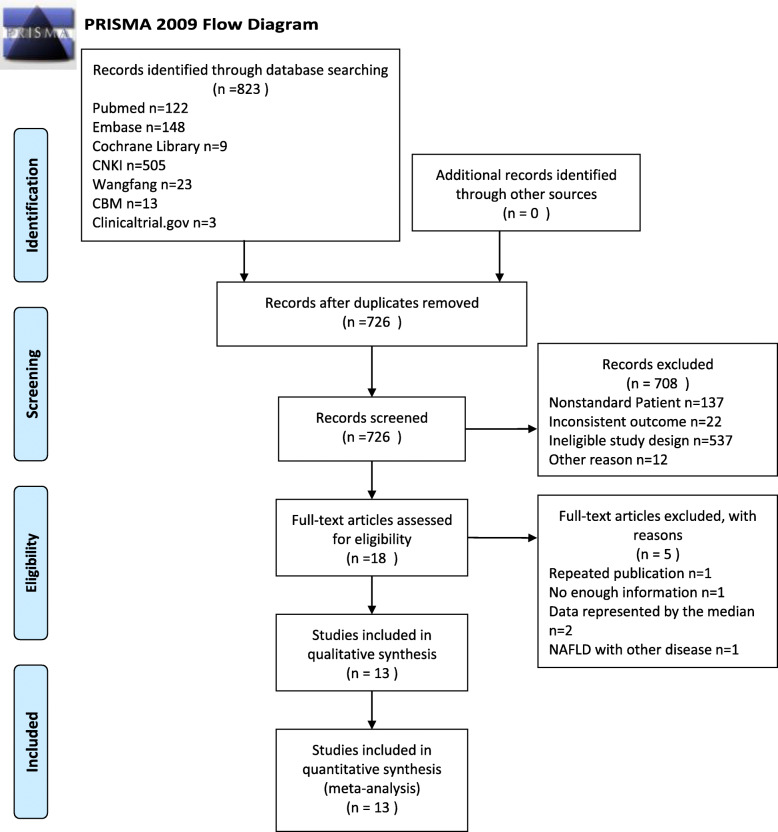
Flowchart of study inclusions and exclusions

The study was completed in accordance with the PRISMA statement. After searching of the seven databases, a total of 823 articles were retrieved. Two researchers screened the literature independently, and any dispute was resolved by a third researcher. Finally, 13 articles (854 cases and 610 controls) were included (Table 1). The overall process is displayed in the form of a PRISMA 2009 flow diagram in Fig. 1. Six articles originated from Chinese databases and seven from English databases. In total, there were nine studies performed in China, two in Turkey, one in South Korea, and one in Germany. All the studies were case-control studies. The age of NAFLD patients is about 35–65 years old and the sex ratio (male%) is 28.85–62.5%. Meanwhile, the age range of healthy people is the same as NAFLD patients’ and the sex ratio (male%) is 31.25–78.43%. In terms of BMI, the BMI of NAFLD patients range from 22 to 30 kg/m^2^, while the BMI of healthy people is about 22–28 kg/m^2^. More detailed characteristics of these studies are listed in Table 1.

**Table 1:** Baseline characteristics of studies included in the meta-analysis

Study	Country	Sample	NAFLD					Control					Method of ANGPTLs measurement	Diagnostic methods	Adjusted factors	Kind of ANGPTLs
			n	ANGPTLs	Sex (male%)	Age	BMI (kg/m^**2**^)	n	ANGPTLs	Sex (male%)	Age	BMI (kg/m^**2**^)				
Gao et al. 2019 [28]	China	serum	180	275.57 ± 22.38(pg/ml)	49.40%	61.25 ± 3.38	27.18 ± 2.77	72	250.23 ± 20.06(pg/ml)	55.56%	60.22 ± 4.24	23.22 ± 1.46	Elisa	ultrasound or Liver biopsy	NA	ANGPTL8
Hong et al. 2017 [29]	China	serum	18	765 ± 301(pg/ml)	38.89%	56.3 ± 4.9	23.4 ± 2.1	12	742 ± 252(pg/ml)	50%	52.2 ± 4.8	23.6 ± 1.7	Elisa	MRI (HCL)	NA	
			18	1129 ± 351(pg/ml)	44.44%	51.9 ± 7.2	26.8 ± 2.4									
Lee et al. 2016 [30]	South Korea	serum	20	1197 ± 638(pg/ml)	\	\	\	18	797 ± 506(pg/ml)	\	\	23.4 ± 3.3	Elisa	ultrasound or CT	age and sex	
Loeffelholz et al. 2017 [31]	Germany	plasma	24	1213.9 ± 203.5(pg/ml)	45.83%	60 ± 3	26.076 ± 4.716	16	1016.5 ± 191.1(pg/ml)	31.25%	54 ± 4	23.8 ± 0.9	Elisa	Liver biopsy	NA	
Long et al. 2019 [32]	China	serum	50	1590 ± 820(pg/ml)	54%	42.62 ± 5.27	25.18 ± 1.54	50	580 ± 520(pg/ml)	50%	42.50 ± 5.38	22.76 ± 0.82	Elisa	ultrasound or Liver biopsy	NA	
Yang et al. 2017 [33]	China	serum	76	1320 ± 620(pg/ml)	56.58%	39.65 ± 10.58	26.87 ± 3.04	68	900 ± 570(pg/ml)	55.88%	40.23 ± 10.72	27.09 ± 3.12	Elisa	ultrasound or Liver biopsy	age and sex	
Zhang et al. 2019 [34]	China	serum	24	387 ± 128.64(pg/ml)	58%	42.86 ± 9.84	22.9 ± 5.64	50	326 ± 102.62(pg/ml)	52%	42.42 ± 9.48	23.54 ± 5.84	\	ultrasound or Liver biopsy	NA	
			58	429 ± 140.84(pg/ml)	(*n* = 100)	(n = 100)	(n = 100)									
			18	585 ± 214.62(pg/ml)												
Zhang et al. 2021 [35]	China	serum	347	1420 ± 670(pg/ml)	56.20%	54.24 ± 5.13	\	120	810 ± 390(pg/ml)	57.50%	55.02 ± 4.78	\	Elisa	ultrasound or Liver biopsy	NA	
Zhu et al. 2016 [36]	China	serum	21	1217.42 ± 427.238(pg/ml)	52.38%	56.00 ± 14.29	26.076 ± 4.716	92	730.03 ± 431.1(pg/ml)	65.22%	53.10 ± 10.06	24.4 ± 3	Elisa	ultrasound or Liver biopsy	age,sex and BMI	
Altun et al. 2018 [37]	Turkey	serum	51	303 ± 286(ng/ml)	\	37.9 ± 9.9	29.2 ± 5.2	30	369 ± 243(ng/ml)	\	34.8 ± 9.5	27.8 ± 4.9	Elisa	ultrasound	NA	ANGPTL4
Yang et al. 2020 [38]	China	serum	28	160 ± 89.86(ng/ml)	\	37.52 ± 4.48	27.69 ± 3.33	47	151.5 ± 125.2(ng/ml)	\	42.83 ± 4.66	23.32 ± 3.4	Elisa	ultrasound	NA	
			24	214.7 ± 104.5(ng/ml)		(*n* = 52)	(n = 52)									
Ma et al. 2019 [39]	China	serum	52	410.4 ± 21.17(ng/ml)	28.85%	37.52 ± 4.48	27.69 ± 3.33	51	582.9 ± 28.07(ng/ml)	78.43%	42.83 ± 4.66	23.32 ± 3.4	Elisa	ultrasound	NA	ANGPTL3
Yilmaz et al. 2009 [40]	Turkey	serum	40	389 ± 110(ng/ml)	62.50%	47.9 ± 11.7	30.5 ± 4.2	14	291 ± 78(ng/ml)	42.86%	45.9 ± 10.1	25.5 ± 2.9	Elisa	ultrasound or Liver biopsy	age and sex	
			8	433 ± 70(ng/ml)	50%	47.3 ± 10.7	28.8 ± 2.7									
			9	321 ± 119(ng/ml)	44.44%	49.1 ± 11.4	29.5 ± 3.7									

Rank by the beginning letter of the first authors. *Abbreviations*: *NAFLD* Non-alcoholic fatty liver disease, *ANGPTLs* Angiopoietin-like proteins, *BMI* Body Mass Index

### Quality assessment

The quality of the studies was evaluated using the NOS score. Their average score was 5.85 in Table 2, which means the whole risk of bias was moderate. All the studies, except those by von Loeffelholz et al. [31] and Zhang et al. [34], had a score of 6; the study by von Loeffelholz et al. [31] lacked representation, and that by Zhang et al. [34] was inadequate in ascertainment of exposure.

**Table 2:** Newcastle-Ottawa Scale (NOS) score of included articles

Number	Author	Year	Selection				Comparability	Exposure			Total	Average
			Adequate definition	Representativeness	Selection of Controls	Definition of Controls		Ascertainment of exposure	Same method	Non-Response rate		
1	Gao et al. [28]	2019	1	1	1	1	0	1	1	0	6	5.85
2	Hong et al. [29]	2017	1	1	1	1	0	1	1	0	6	
3	Lee et al. [30]	2016	1	1	1	1	0	1	1	0	6	
4	Loeffelholz et al. [31]	2017	1	0	1	1	0	1	1	0	5	
5	Long et al. [32]	2019	1	1	1	1	0	1	1	0	6	
6	Yang et al. [33]	2017	1	1	1	1	0	1	1	0	6	
7	Zhang et al. [34]	2019	1	1	1	1	0	0	1	0	5	
8	Zhang et al. [35]	2021	1	1	1	1	0	1	1	0	6	
9	Zhu et al. [36]	2016	1	1	1	1	0	1	1	0	6	
10	Altun et al. [37]	2018	1	1	1	1	0	1	1	0	6	
11	Yang et al. [38]	2020	1	1	1	1	0	1	1	0	6	
12	Ma et al. [39]	2019	1	1	1	1	0	1	1	0	6	
13	Yilmaz et al. [40]	2009	1	1	1	1	0	1	1	0	6	

Overall certainty of evidence evaluated by the GRADE system was assessed as very low in Supplementary Additional file 3. Observational studies make the certainty of evidence start from low. The certainty of evidence is gradually degraded owing to serious risk of bias, serious inconsistency and serious inprecision (ANGPTL3 and ANGPTL4). The low NOS scores (5–6) are responsible for serious risk of bias. Serious inconsistency is due to the significant heterogeneity (I^2^>40%) among the included articles. For ANGPTL3 and ANGPTL4, the sample size is relatively small (*n*<400), accounting for serious inprecision. Meanwhile, some articles do not strictly match the basic information of NAFLD patients and healthy people, which also affects the certainty of evidence.

### Association between the ANGPTLs level and NAFLD

**Fig. 2:**
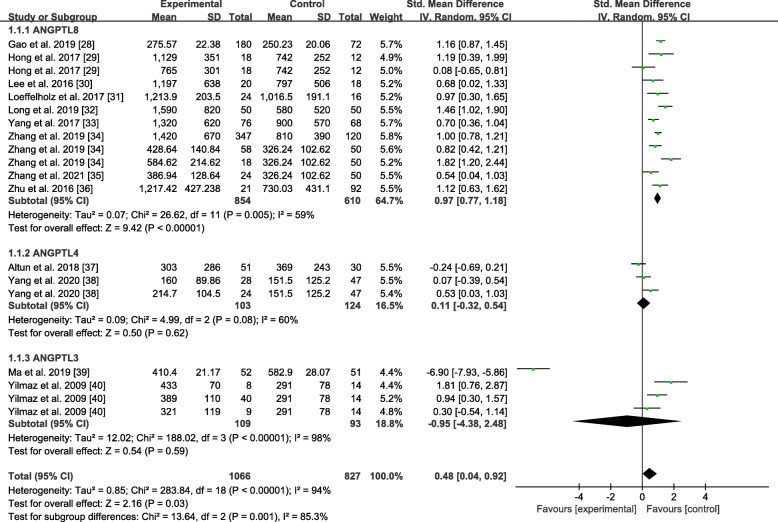
Forest plot of circulating ANGPTLs levels between NAFLD and the healthy control group (Random-Effects Model, SMD)

**Fig. 3:**
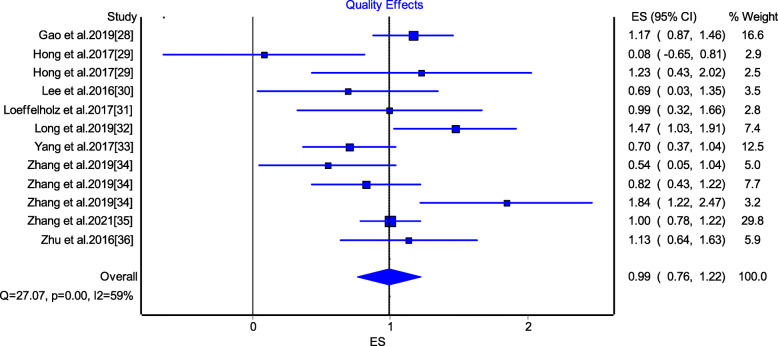
Forest plot of circulating ANGPTL8 levels between NAFLD and the healthy control group (Quality-Effects Model, SMD)

Figure 2 shows the ANGPTLs levels between the NAFLD group and the the healthy control group. Because of the high heterogeneity, the random-effects model was selected. In this model, the overall SMD was 0.48 (95%CI: 0.04, 0.92). The ANGPTL8 level was significantly higher in the NAFLD group than in the healthy control group, whose SMD was 0.97 (95%CI: 0.77, 1.18); conversely, there was no significant difference in the ANGPTL 4 (SMD = 0.11, 95%CI: − 0.32, 0.54) and ANGPTL3 levels (SMD = − 0.95, 95%CI: − 4.38, 2.48) between the two groups. The analysis demonstrated that the NAFLD group had a significantly higher ANGPTL8 level than the healthy control group. To reduce the impact of heterogeneity on the results, the quality-effects model for those related to the ANGPTL8 level was further utilised, as shown in Fig. 3. In this model, the analysis also demonstrated that the ANGPTL8 level in the NAFLD group was still significantly higher than that in the healthy control group (SMD = 0.99, 95%CI: 0.76, 1.22).

**Fig. 4:**
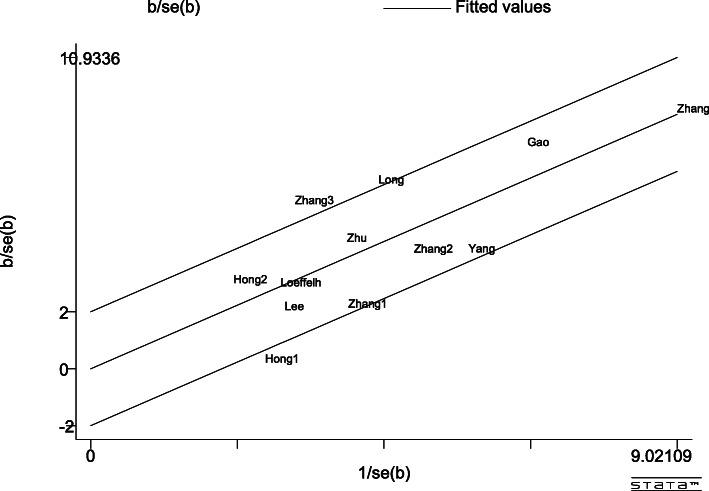
Galbraith Test result of circulating ANGPTL8 levels between NAFLD and the healthy control group

There was a certain heterogeneity in the ANGPTL8 level obtained using the Galbraith test among the studies included, as shown in Fig. 4. Most of the articles had levels within reasonable ranges. Furthermore, subgroup and meta-regression analysis were performed to identify the sources of heterogeneity.

#### Subgroup analysis by race

**Fig. 5:**
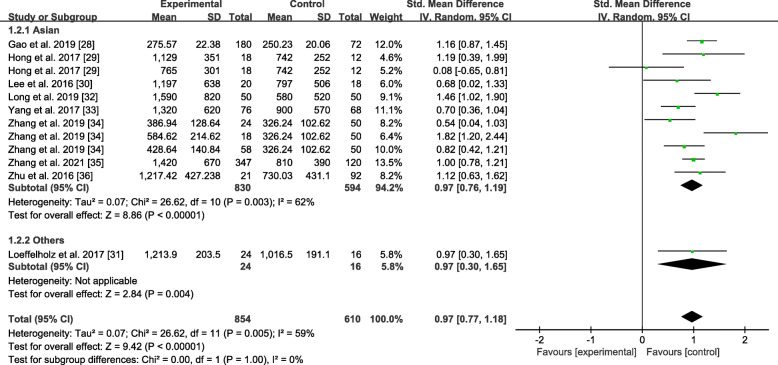
Forest plot of circulating ANGPTL8 levels between NAFLD and the healthy control group by race (Random-Effects Model, SMD)

The results of the subgroup analysis of the studies classified by race are shown in Fig. 5. The Asian study subgroup presented high heterogeneity (*P* = 0.003, I^2^ = 62%), and the overall SMD was 0.97 (0.76, 1.19). Moreover, among this subgroup, the NAFLD group had higher ANGPTL8 levels than the healthy control group. Among the other race subgroups, the result seemed to be similar to the overall SMD of 0.97 (0.30, 1.18). The ANGPTL8 levels between the NAFLD group and the healthy control group also showed significant differences in other races.

#### Subgroup analysis by severity

**Fig. 6:**
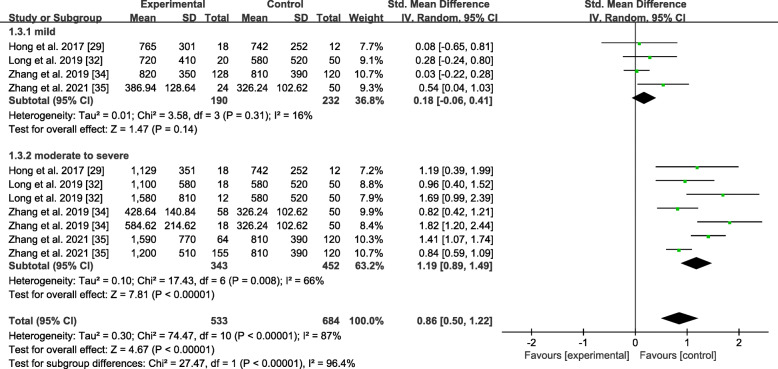
Forest plot of circulating ANGPTL8 levels between NAFLD and the healthy control group by severity (Random-Effects Model, SMD)

As shown in Fig. 6, the random-effects model was applied to this subgroup analysis. The ANGPTL8 levels were not significantly different between the mild NAFLD group and the healthy control group with an overall SMD of 0.18 (− 0.06, 0.41). On the contrary, there was a significant difference between the moderate to severe NAFLD group and the healthy control group with an overall SMD of 1.19 (0.89, 1.49). The moderate to severe NAFLD group seemed more likely to have higher ANGPTL8 levels than the mild NAFLD group.

#### Subgroup analysis by BMI

**Fig. 7:**
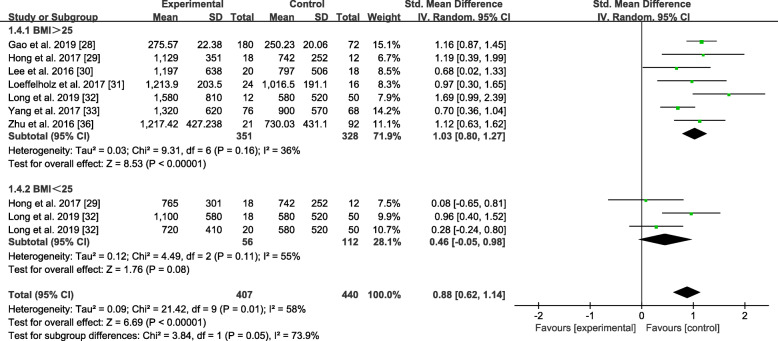
Forest plot of circulating ANGPTL8 levels between NAFLD and the healthy control group by BMI (Random-Effects Model, SMD)

The results of the subgroup analysis of the studies classified by BMI are shown in Fig. 7. The NAFLD group was divided into the BMI > 25 kg/m^2^ and BMI < 25 kg/m^2^ subgroups. As the statistics showed, the BMI > 25 kg/m^2^ subgroup had a significant tendency to have high ANGPTL8 levels with an overall SMD of 1.03 (0.80, 1.27). Meanwhile, the ANGPTL8 levels in the BMI < 25 kg/m^2^ subgroup did not significantly differ from those in the healthy control group. The overall SMD was 0.46 (− 0.05, 0.98).

#### Robust error meta-regression analysis

A meta-regression analysis that adjusted for the effect of the two groups’ BMI was performed. Based on the robust error meta-regression analysis findings, it can be concluded that the BMI might well explain the high heterogeneity (*P* < 0.05); the SMD was 0.06 (0.002, 1.39) when the influence of BMI was zero.

### Sensitivity analysis

**Fig. 8:**
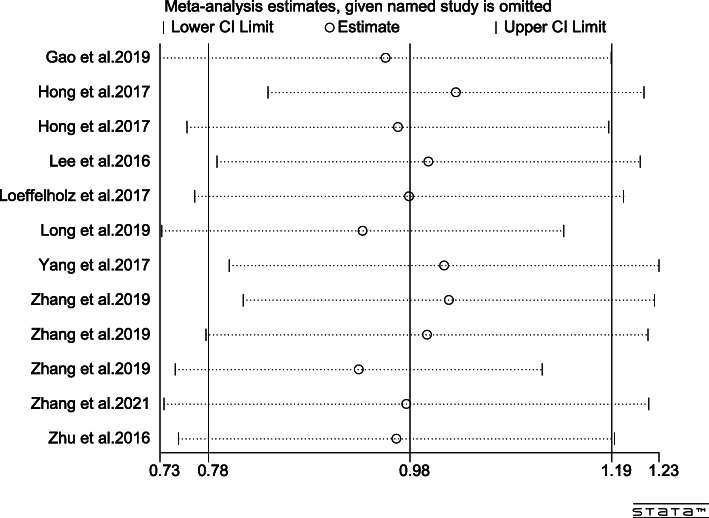
Omission of single study figure

**Fig. 9:**
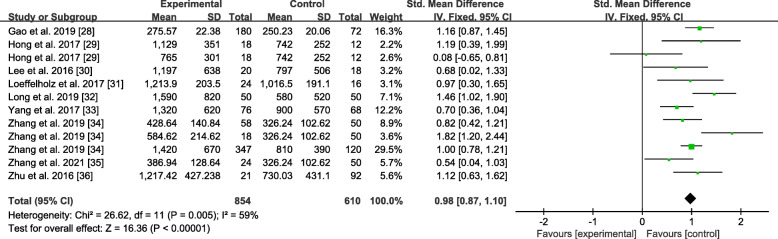
Forest plot of circulating ANGPTL8 levels between NAFLD and the healthy control group (Fixed-Effects Model, SMD)

**Fig. 10:**
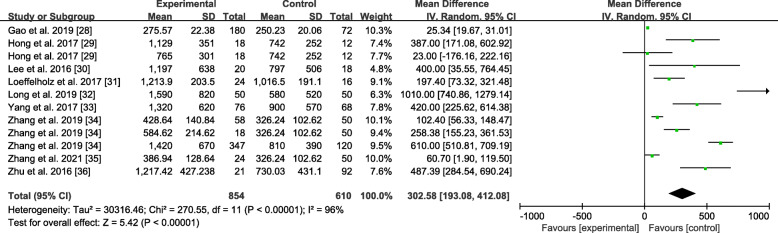
Forest plot of circulating ANGPTL8 levels between NAFLD and the healthy control group (Random-Effects Model, MD)

Omission of a single study was performed to evaluate the sensitivity of the study results in ANGPTL8 levels, as shown in Fig. 8. By excluding one of the articles, a rough idea of the impact of this article on the overall results could be understood. No particular article was found to have a significant impact on the outcomes. The results were invariant when the fixed-effects model was selected with SMD = 0.98 (0.87, 1.10) (Fig. 9). If MD were applied, the same result would still be obtained in Fig. 10: MD = 302.58 (193.08, 412.08).

### Publication bias

**Fig. 11:**
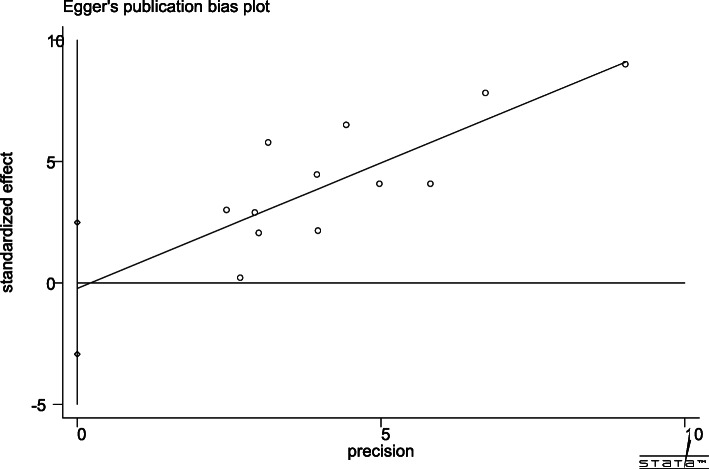
Egger’s publication bias plot

**Fig. 12:**
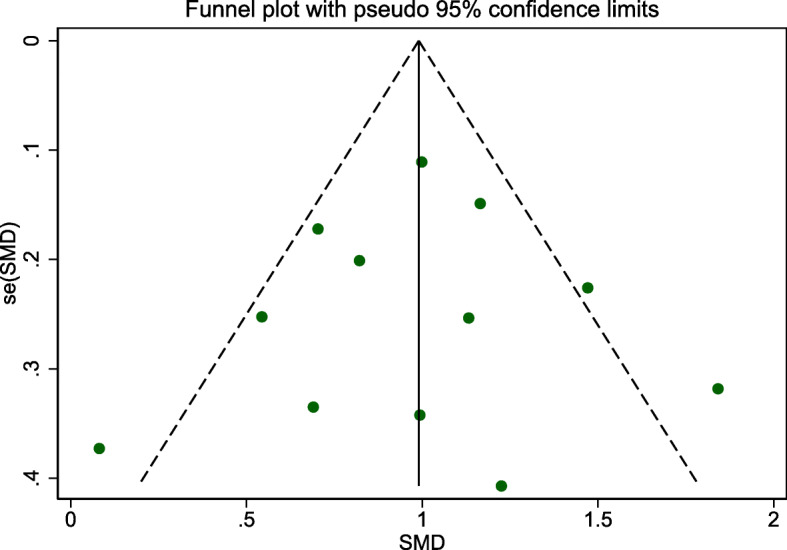
Funnel figure

**Fig. 13:**
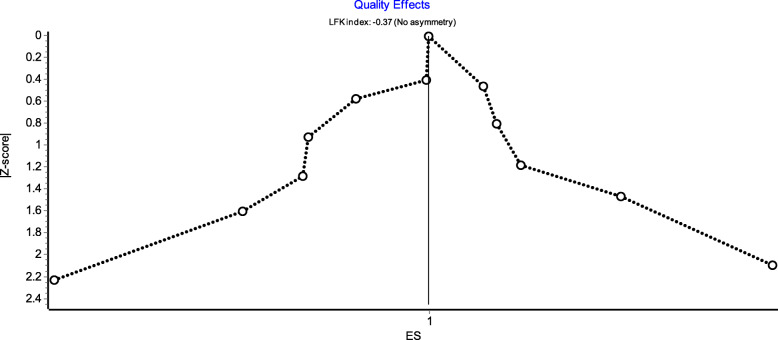
Doi plot

Egger’s publication bias plot is shown in Fig. 11. The total publication bias analysis performed using Stata 16 suggested a low possibility of publication bias (*P* = 0.86 > 0.10). The funnel figures are shown in Fig. 12. The overall figure was symmetric, which also indicated a low possibility of publication bias. The entire study presented no asymmetry of the study using the Doi plot (LFK index = − 0.37) (Fig. 13).

## Discussion

Recently, an increasing number of clinical investigators have found the nutritional, hormonal, and molecular regulations of ANGPTLs in some metabolic tissues, such as the liver. Tikka et al. [41] found that the liver-specific mechanism is correlated with the insulin-sensitive phenotype of ANGPTL3-deficient humans. Dikker et al. [42] believed that ANGPTL4 regulates plasma lipoprotein levels by inhibiting LPL activity and thus performed a cohort study to evaluate the relationship between serum ANGPTL4 levels and obesity and hepatic steatosis in adolescents. Zhang et al. [43] demonstrated that insulin can affect the expression of ANGPTL8 in hepatocytes and adipocytes in mice.

NAFLD is a manifestation of multisystem metabolic disorders involving the liver, whose prevalence is rising to alarming levels [44]. In a sense, NAFLD is considered both a cause and a result of a metabolic disorder, forming a vicious circle [45], which ultimately leads to the progression of a series of metabolic diseases [46]. Unhealthy diet and irregular lifestyle lead to an increasing prevalence of the disease annually, which affects the daily lives of affected patients and is more likely to cause other cardiovascular and cerebrovascular diseases [47, 48]. However, there is still a lack of effective diagnostic methods for this disease. Although imaging can provide some assistance, it is not used routinely and carries a radiation risk. Liver biopsy can also induce further pain. Therefore, it is of great clinical value to measure the prevalence and severity of NAFLD using common blood sampling methods.

In the study, 823 articles were initially found by searching seven databases; 726 articles remained after the exclusion of repeated articles, and 13 articles were finally included, consisting of 9 articles from China, 2 from Turkey, 1 from South Korea, and 1 from Germany. The pooled meta-analysis showed that some ANGPTLs may be closely related to NAFLD and that the ANGPTL8 level is significantly higher in patients with NAFLD than in healthy individuals. The quality-effects model also demonstrated that the ANGPTL8 level was significantly higher in the patients with NAFLD. In contrast, no remarkable results were obtained for ANGPTL3 and ANGPTL4 owing to the limited amount of research available. Therefore, the relationship between ANGPTL8 and NAFLD has only been discussed.

High heterogeneity was observed in the study; thus, the multiple subgroup analyses by race, severity and BMI have been conducted to identify the sources. With regard to race, the ANGPTL8 levels between the Asians and other races with NAFLD showed no significant difference, while the levels were both higher than those in the healthy control group. In terms of severity, the patients with moderate to severe NAFLD presented slightly higher ANGPTL8 levels than the patients with mild NAFLD; however, there was still no significant difference observed. In terms of BMI, a similar result was obtained. The patients with NAFLD with a BMI of > 25 kg/m^2^ had higher levels of circulating ANGPTL8, while those with a BMI of < 25 kg/m^2^ and the healthy controls seemed to have no difference in the ANGPTL8 level. As many studies did not strictly match the BMI of the patient and the healthy control groups, a robust error meta-regression analysis was performed to measure the results after BMI adjustment. The BMI was the source of heterogeneity. The ANGPTL8 levels in the patients with NAFLD were still significantly high even after the BMI of the different groups was adjusted. There might still be many factors that affect the significantly high levels of ANGPTL8 in patients with NAFLD, such as its severity.

In addition, the omission of a single study for the sensitivity analysis showed a robust result. No significant changes occurred after any of the included articles were excluded. Similarly, no significant change in the results occurred even when the fixed-effects model or MD was applied to the study. Based on the findings of Egger’s test and funnel figure, there was no obvious publication bias in this study, and most articles had values within a reasonable range. Meanwhile, the Doi plot appeared to have no asymmetry, which means small study effects were low in this study. All of these proved that the results are representative to some degree. However, it’s hard to ignore the very low certainty of evidence, according to the GRADE system.

### Study strengths and limitations

The study covers clinical research on ANGPTLs and NAFLD in the past 15 years. The general relationship between ANGPTLs and NAFLD can be understood by summarising the information on all aspects. ANGPTLs may act as a key indicator for the diagnosis of NAFLD, which is generally difficult to diagnose and treat in the early stages. It might not only reduce the suffering of patients but also save medical resources. Simultaneously, the study also found that ANGPTL8 could reflect the progress of NAFLD to some extent, which is of great significance for disease progress detection.

However, there are still some limitations to the study. First, the number of articles and cases eventually included was small; thus, the evidence to draw conclusions was weak. Although there have been many studies conducted on the relationship between NAFLD and circulating ANGPTLs, most of them also included other metabolic syndromes, such as diabetes. To avoid the influence of confounding factors, patients with NAFLD were included only. Consequently, the overall number of cases included was relatively small, only 1066 cases (ANGPTL8: 854 cases, ANGPTL4: 103 cases, ANGPTL3: 109 cases). The results were only inferred from a limited number of studies, which may be one-sided. Secondly, the included articles originated mostly from Asia. Twelve studies originated from Asia, while only the study of Loeffelholz et al. [31] came from Europe; therefore, whether the results are applicable to other areas is uncertain. Although four English databases have been searched already, the research in other areas is relatively less and greater in the form of quartiles. Therefore, more datasets in other areas are required for a comprehensive conclusion. Thirdly, the circulating ANGPTLs level units obtained from the different studies were not uniform. Even if the study had been converted to the same unit, the values varied considerably. For NAFLD patients, the highest value of ANGPTL8 was up to 1590 pg/ml (Long et al’s [32]), while the lowest was only 275.57 pg/ml (Gao et al’s [28]). Consequently, the SMD was used in this meta-analysis. Fourthly, the results showed some heterogeneity, which may be attributed to the different centre settings, time limitations, race, and other reasons. Moreover, there may be obvious sources of bias in each study. Although most studies strictly controlled for factors, such as age and sex, there may still be many unexpected factors. At the same time, the study indicates that BMI may be the source of heterogeneity, but there is inevitably a certain risk of ecological bias because of the use of aggregate data of patients other than individual participant data. Therefore the results should be interpreted with caution. Finally, ANGPTLs are considered to be a family of lipids, and insulin resistance is closely related to them. From the perspective of NAFLD, studies on the insulin resistance index (HOMA-IR) are of great clinical value. However, as most of the included articles did not involve the HOMA-IR [28, 32, 33, 35, 38, 39], a reasonable subgroup analysis could not be performed. Therefore, this was not discussed in this study.

## Conclusions

In summary, this meta-analysis demonstrated that ANGPTLs may be closely related to NAFLD and that the ANGPTL8 level is significantly higher in patients with NAFLD than in healthy individuals. ANGPTL8 may be an emerging biomarker for NAFLD. As the article pool used in this study was relatively small and biased to certain regions, the exact relationship between other ANGPTLs and NAFLD needs further exploration. Furthermore, because of the very low certainty evaluated by the Grade system, it’s necessary to interpret the results more carefully. Therefore, in the future, more regional, multi-aspect, and multi-centre studies are required to determine the role of ANGPTLs as markers for the diagnosis of NAFLD and to clarify whether ANGPTL8 can be a highly sensitive indicator in different stages of NAFLD.

## Supplementary Information

**Additional file 1.** PRISMA 2009 checklist

**Additional file 2.** PROSPERO: Number CRD42020159432.

**Additional file 3.** GRADE summary of findings table.

## Data Availability

All data generated or analysed during this study are included in this published article and its supplementary information files.

## Abbreviations

ANGPTLs: Angiopoietin-like proteins
ANGPTL 1–8: Angiopoietin-like 1–8
NAFLD: Non-alcoholic fatty liver disease
MAFLD: Metabolic-associated fatty liver disease
NASH: Non-alcoholic steatohepatitis
SMD: Standardized Mean Difference
CI: Confidence intervals
NOS: Newcastle-Ottawa Scale
BMI: Body Mass Index
LPL: Lipoprotein lipase
