# Supplementary material for: Circulating angiopoietin-like proteins in metabolic-associated fatty liver disease: a systematic review and meta-analysis
Source: Lipids Health Dis. 2021 May 25;20:55. doi: 10.1186/s12944-021-01481-1 (PMC8152125; doi:10.1186/s12944-021-01481-1)
Supplement: Supplementary file 2 — Additional file 2. PROSPERO: Number CRD42020159432. [file 12944_2021_1481_MOESM2_ESM.pdf]

Relationship of circulating betatrophin in patients with Nonalcoholic Fatty Liver disease(NAFLD):a systematic review and meta-analysis

Fangping Wu, Yani Ke

To enable PROSPERO to focus on COVID-19 registrations during the 2020 pandemic, this registration record was automatically published exactly as submitted. The PROSPERO team has not checked eligibility.

Citation

Fangping Wu, Yani Ke. Relationship of circulating betatrophin in patients with Nonalcoholic Fatty Liver disease(NAFLD):a systematic review and meta-analysis. PROSPERO 2020 CRD42020159432 Available from:
https://www.crd.york.ac.uk/prospero/display_record.php?ID=CRD42020159432

Review question

Studies on the relationship between circulating betatrophin and Nonalcoholic Fatty Liver disease

show controversial conclusions, which may confuse researchers.Our systematic review and meta-analysis aim to summarize existing literature and assess the relationship between them.Furthermore, our secondary aim is to analyze the correlation between circulating betatrophin and insulin resistance in patients with Nonalcoholic Fatty Liver disease.

Searches

We will search some databases including PubMed, Cochrane Library, Cochrane Register of Controlled Trials(CENTRAL), EMBASE, CNKI, WANFANG and CBM, using the terms “fatty liver” (OR “NAFLD” OR “nonalcoholic fatty liver disease”) AND "betatrophin" or "ANGPTL8". Furthermore, the reference lists of enrolled studies and publications with citations of included papers will also be reviewed for suitable papers. We will also contact study authors by e-mail to clarify additional studies and ask for missing data. No language limitations will be used.

Types of study to be included

Case-control studies or cohort studies published report data on circulating betatrophin level in individuals with/without NAFLD will be included.

Condition or domain being studied

Nonalcoholic fatty liver disease (NAFLD) is a spectrum of liver disorders . It is a condition defined by the presence of steatosis in more than 5% of hepatocytes with little or no alcohol consumption. NAFLD consists of the benign non-alcoholic fatty liver (NAFL), and the more severe non-alcoholic steatohepatitis (NASH). NASH is a more progressive form of NAFLD and is characterized by steatosis, hepatocellular ballooning, lobular inflammation and almost always fibrosis. In an effort to regenerate new cells, NASH progresses to cirrhosis with the hepatocytes replaced by scar tissues of type I collagen produced by stellate cells. Cirrhosis is an end-stage organ failure that require liver transplantation or may lead to hepatocellular carcinoma.

Participants/population

?We will include only studies conducted in adults (aged 18 years or older) who were diagnosed as NAFLD either by imaging or by histology.

?We will exclude those including secondary hepatic fat accumulation, such as significant alcohol nsumption, use of steatogenic medication, or hereditary disorders, other known causes of liver diseases, eg virus and drugs.

Intervention(s), exposure(s)

Individuals with NAFLD, Circulating betatrophin level , insulin resistance index.

Comparator(s)/control

Control cases will be healthy people.In addition, patients with different levels of NAFLD will also be considered.

Main outcome(s)
Main outcome is circulating betatrophin level in either plasma or serum.

* Measures of effect

None

Additional outcome(s)
The relationship between circulating betatrophin and insulin resistance in patients with Nonalcoholic Fatty Liver Disease.

* Measures of effect

None

Data extraction (selection and coding)
Two of the researchers will independently extract data.With the help of one researcher, we will reach a common regarding including first author's last name, publication date, country of origin, the Newcastle-Ottawa Scale (NOS), numbers of cases and controls, betatrophin levels measured method, levels of betatrophin in NAFLD group/ control group, levels of insulin resistance indexes in NAFLD group/ control group.

Risk of bias (quality) assessment
We will evaluate the quality of the included articles by using Newcastle-Ottawa Scale (NOS) scale. The NOS consists of three dimensions including selection, comparability, and exposure which reflect in eight items. For each item, there are a series of response options . A star system is used to allow a semi-quantitative assessment of study quality.For example, the highest quality studies will be awarded a maximum of one star for each item except for those related to comparability that allows two stars. The NOS ranges from zero to nine stars.

Strategy for data synthesis
Review manager 5.3 and Stata 15 software will be used for statistical analysis to perform meta-analysis.Heterogeneity will be checked by the ?^{2} test and the I^2 statistic. The criteria for identification of heterogeneity will be a P value less than 0.10 for the ? ^{2}test and an I^2statistic greater than 50%.

Analysis of subgroups or subsets
Subgroup analyses will be performed by Severity of NAFLD .

Contact details for further information
Fangping Wu
201512201504001@zcmu.edu.cn

Organisational affiliation of the review
Zhejiang Chinese Medical University

Review team members and their organisational affiliations
Miss Fangping Wu. Zhejiang Chinese Medical University
Miss Yani Ke. Zhejiang Chinese Medical University

Type and method of review
Meta-analysis, Systematic review

Anticipated or actual start date
NaN undefined NaN

Anticipated completion date
NaN undefined NaN

Funding sources/sponsors
None

Conflicts of interest
None known

Language
English

Country
China

Stage of review
Review Ongoing

Subject index terms status
Subject indexing assigned by CRD

Subject index terms
MeSH headings have not been applied to this record

Date of registration in PROSPERO
28 April 2020

Date of first submission
21 November 2019

Stage of review at time of this submission
The review has not started

Stage	Started	Completed
Preliminary searches	No	No
Piloting of the study selection process	No	No
Formal screening of search results against eligibility criteria	No	No
Data extraction	No	No
Risk of bias (quality) assessment	No	No
Data analysis	No	No

The record owner confirms that the information they have supplied for this submission is accurate and complete and they understand that deliberate provision of inaccurate information or omission of data may be construed as scientific misconduct.

The record owner confirms that they will update the status of the review when it is completed and will add publication details in due course.

Versions
28 April 2020

PROSPERO
This information has been provided by the named contact for this review. CRD has accepted this information in good faith and registered the review in PROSPERO. The registrant confirms that the information supplied for this submission is accurate and complete. CRD bears no responsibility or liability for the content of this registration record, any associated files or external websites.
