# Supplementary material for: Circulating angiopoietin-like proteins in metabolic-associated fatty liver disease: a systematic review and meta-analysis
Source: Lipids Health Dis. 2021 May 25;20:55. doi: 10.1186/s12944-021-01481-1 (PMC8152125; doi:10.1186/s12944-021-01481-1)
Supplement: Supplementary file 3 — Additional file 3. GRADE summary of findings table. [file 12944_2021_1481_MOESM3_ESM.pdf]

GRADE summary of findings table.

Outcome	Participants (studies)	Risk of bias	Inconsistency	Indirectness	Imprecision	Other considerations	Overall certainty of evidence
ANGPTL8	9 observational studies	Serious[1]	Serious[2]	Not serious	Not serious	Plausible confounding[4]	⊕OOO VERY LOW
ANGPTL4	2 observational studies	Serious[1]	Serious[2]	Not serious	Serious[3]	Plausible confounding[4]	⊕OOO VERY LOW
ANGPTL3	2 observational studies	Serious[1]	Serious[2]	Not serious	Serious[3]	Plausible confounding[4]	⊕OOO VERY LOW

[1]Serious risk of bias due to the NOS score (5-6)

[2]Serious inconsistency due to significant heterogeneity (I^2＞40%)

[3]Serious imprecision due to small sample size (n＜400)

[4]all plausible residual confounding would reduce the demonstrated effect. Some of the studies do not match the basic information of two groups, like age, BMI and so on.
